# A crisis planning and monitoring intervention to reduce compulsory hospital readmissions (FINCH study): protocol for a randomised controlled feasibility study

**DOI:** 10.1186/s40814-024-01453-z

**Published:** 2024-02-20

**Authors:** Sonia Johnson, Mary Birken, Patrick Nyikavaranda, Ariana Kular, Rafael Gafoor, Jordan Parkinson, Chloe Hutchings-Hay, Thomas Gant, Jazmin Molai, Jessica Rivera, James Fenwick, Caroline Bendall, Louise Blakley, Theresa Bacarese-Hamilton, Valerie Christina White, Mark Keith Holden, Janet Seale, Jackie Hardy, Kathleen Lindsay Fraser, Lizzie Mitchell, Barbara Lay, Henrietta Mbeah-Bankas, Paul McCrone, Nick Freemantle, Lisa Wood, Fiona Lobban, Brynmor Lloyd-Evans

**Affiliations:** 1 Division of Psychiatry, Faculty of Brain Sciences, University College London, 6Th Floor, 149 Tottenham Court Road, London, W1T 7NF UK; 2 Camden and Islington NHS Foundation Trust, London, UK; 3 Department of Primary Care & Public Health, Brighton & Sussex Medical School, University of Sussex, Brighton, UK; 4 Comprehensive Clinical Trials Unit, University College London, London, UK; 5 Lancashire and South Cumbria NHS Foundation Trust, Preston, UK; 6 South London and Maudsley NHS Foundation Trust, London, UK; 7 North East London NHS Foundation Trust, London, UK; 8 Institute of Psychiatry, Psychology and Neuroscience, Kings College London, London, United Kingdom; 9 Southern Health NHS Foundation Trust, Calmore, UK; 10 Lucerne Psychiatry, Lucerne, Switzerland; 11 NHS England, London, UK; 12 Institute for Lifecourse Development, University of Greenwich, London, UK; 13 Faculty of Health and Medicine, Lancaster University, Lancaster, United Kingdom

**Keywords:** Involuntary admission, Compulsory detention, Crisis planning, Self-management, Co-production

## Abstract

**Background:**

Rates of compulsory (also known as involuntary) detention under mental health legislation have been rising over several decades in countries including England. Avoiding such detentions should be a high priority given their potentially traumatic nature and departure from usual ethical principles of consent and collaboration. Those who have been detained previously are at high risk of being detained again, and thus a priority group for preventive interventions. In a very sparse literature, interventions based on crisis planning emerge as having more supporting evidence than other approaches to preventing compulsory detention.

**Method:**

We have adapted and manualised an intervention previously trialled in Zürich Switzerland, aimed at reducing future compulsory detentions among people being discharged following a psychiatric admission that has included a period of compulsory detention. A co-production group including people with relevant lived and clinical experience has co-designed the adaptations to the intervention, drawing on evidence on crisis planning and self-management and on qualitative interviews with service users and clinicians. We will conduct a randomised controlled feasibility trial of the intervention, randomising 80 participants to either the intervention in addition to usual care, or usual care only. Feasibility and acceptability of the intervention and trial procedures will be assessed through process evaluation (including rates of randomisation, recruitment, and retention) and qualitative interviews. We will also assess and report on planned trial outcomes. The planned primary outcome for a full trial is repeat compulsory detention within one year of randomisation, and secondary outcomes include compulsory detention within 2 years, and symptoms, service satisfaction, self-rated recovery, self-management confidence, and service engagement. A health economic evaluation is also included.

**Discussion:**

This feasibility study, and any subsequent full trial, will add to a currently limited literature on interventions to prevent involuntary detention, a goal valued highly by service users, carers, clinicians, and policymakers. There are significant potential impediments to recruiting and retaining this group, whose experiences of mental health care have often been negative and traumatising, and who are at high risk of disengagement.

**Trial registration:**

ISRCTN, ISRCTN11627644. Registered 25th May 2022, https://www.isrctn.com/ISRCTN11627644.

**Supplementary Information:**

The online version contains supplementary material available at 10.1186/s40814-024-01453-z.

## Background

Compulsory detentions in mental health inpatient units have been increasing over several decades in England, as in several other European countries [1]. In England, the Mental Health Act (MHA) came into force in 1983 and has subsequently been amended but not so far replaced: an Independent Review published in 2018 called for considerable change [2], with recommendations accepted by the Government but not so far enacted in law as of February 2023. It allows for compulsory detention in hospitals on grounds of risk to self or others or deteriorating health, although the latter criterion is used less than those based on risk-based criteria. Orders that can be made through the MHA are for a 28-day period of detention in the hospital for assessment (Section 2) and for a 6-month period for treatment (Section 3), both agreed upon by two suitably qualified doctors and an Approved Mental Health Professional, a professional (often a social worker) who is not a doctor and has specific training in implementing the Act. Official data suggest the use of the Mental Health Act to detain people in hospitals increased by 40% between 2006 and 2016 [2], with further yearly rises since 2016, when the method for enumerating admissions was changed [3]. There is also a striking ethnic inequality in risk of being detained, with people from Black and Black British ethnic groups around four times as likely to be detained as White British people [2–5].

High rates of compulsory admission are an important problem because service users and carers recurrently report that this is a distressing and traumatising experience that greatly disrupts recovery and therapeutic alliances [6, 7]. Compulsory detention, and the coercion and disenfranchisement that are necessarily involved, also violate an otherwise highly regarded principle that mental health treatment should be freely chosen and as collaborative as possible. Thus, there is a strong case for keeping compulsory (also known as involuntary) admissions to a minimum. The experiences of ethnic minority communities are especially important, as high rates of coercive treatment, especially in Black/Black British communities, constitute an important inequality and contribute to mistrust of mental health services and thus to disengagement [8]. Compulsory admissions are also expensive, recently estimated as costing an average of £18,315 per admission [3], with limited clinical or social gains evident at 1 year follow-up [9]. Policymakers and service user advocates thus concur in prioritising the prevention of compulsory admission.

Currently, we lack strategies for preventing compulsory admission that are evidence-based and have been successfully implemented as part of standard mental health care in the UK or elsewhere: there are surprisingly few published trials of interventions with compulsory admission as a primary, or even as a secondary outcome measure [10]. There is considerable evidence that a group at high risk of compulsory admission is those who have already been detained at least once [11], making them a priority for interventions to reduce further compulsory detention. One approach has been the continuing compulsion into the community, for example through Compulsory Treatment Orders in England. However, current evidence does not support this as a means of reducing compulsory admissions [10, 12], and there is some evidence of disproportionate use in Black or Black British ethnic groups [3]. When evidence from all available studies internationally is pooled through meta-analysis [13, 14], the only kind of intervention that currently has substantial evidence for effectiveness in reducing compulsory admissions is advance planning for crises (often called crisis plans) and collaborative agreements (advance statements) with patients about what should happen if they are unwell in future. Such strategies were recommended for national roll-out in the Independent Review of the Mental Health Act in England, published in 2018 [4].

Informed by this evidence, our aim in this study is to develop and test in a feasibility study an intervention designed to reduce future compulsory detentions through support including person-centred crisis plans for people who have just had a mental health admission during which they were compulsorily detained. A particular concern, given inequalities in detention and overall experiences of mental health care, is that the intervention should be suitable and engaging for people from ethnic groups that place them at increased risk of being detained, such as people from Black African, Caribbean, and British backgrounds.

In our group’s review of relevant literature [13], we found that while pooled meta-analysis indicates overall effectiveness for interventions based on crisis planning, there has been considerable variation between studies in effect size and in whether statistical significance was reached. Difficulties in implementing crisis planning interventions effectively were noted in several studies. In particular, in the largest UK trial, a crisis planning model that had initially appeared effective in a single-site trial showed little evidence of effectiveness when tested across multiple sites at a larger scale [15–17]. This was attributed to clinicians often failing to modify their routine practice to incorporate crisis planning as intended, and to crisis plans rarely being referred to by clinicians or service users in subsequent care or help-seeking. Thus, it is likely that, to be reliably successful in reducing compulsory admission, crisis planning needs to be embedded in a framework that ensures it is delivered in practice, and that the crisis plans that are formulated are subsequently monitored and followed through.

Within our systematic review [13], we identified one trial as appearing to have a more intensive and developed approach to implementation than the rest, including strategies for continued monitoring for signs of crisis and for giving service users a voice. In this study, carried out in the multicultural Swiss city of Zürich [18, 19], researchers designed and tested a programme of psychoeducation, crisis planning, and monitoring by phone for people being discharged following a compulsory hospital admission. Findings were promising: over 2 years, 28% of people in this programme were compulsorily readmitted compared with 43% of controls receiving standard local care: with adjustment for other differences, the estimated relative risk of compulsory readmission for the treatment group was 0.55 (95% confidence interval 0.33–0.94) [19]. Importantly, the follow-up element of monthly monitoring phone calls by a “personal therapist” additional to the usual care team built in a solution to the problem identified in other studies of crisis plans being neglected and under-used [17]. However, the Zürich trial had some important limitations: it did not achieve the intended statistical power, and differential drop-out rates created ambiguity in interpreting the statistically significant result. Despite this, it seemed sufficiently promising in a field in which robust research is sparse to form a starting point for our programme.

The aim of the FINCH study is to review and adapt the Zürich intervention to a UK context, and to examine the feasibility and acceptability of delivering the intervention and testing it through a randomised controlled trial. In adapting it, we aimed also to incorporate any other relevant evidence on self-management and crisis planning interventions, the perspectives of service users and carers with relevant lived experience and of professionals with relevant clinical experience, and any relevant policy directives and guidance.

Specific objectives of our study are the following:

- Phase 1: intervention development and preliminary testing.
  (i) To adapt and manualise the Zürich crisis planning and monitoring intervention through an iterative co-design process, informed by inputs including qualitative interviews with service users and staff with relevant experience, and relevant evidence on the implementation of crisis planning and self-management, and considering especially the needs and experiences of people from ethnic backgrounds at higher risk of detention (objective already completed).
  (ii) To deliver the intervention to a preliminary group of six participants, allowing us to study their experiences and those of clinicians delivering the intervention, and to refine the intervention based on this (objective already completed).
- Phase 2: feasibility study.
  i) To test the feasibility and acceptability of the intervention through a feasibility randomised controlled trial and accompanying qualitative study, assessing the feasibility of recruiting, randomising, and retaining over a 2-year period participant, and investigating acceptability via qualitative interviews.
  ii) To assess the difference in a number of participants in each arm of the trial who experience at least one episode of compulsory detention within 12 months of randomisation (proposed primary outcome for a definitive trial), as well as assessing secondary clinical and social outcomes and health economic parameters.
  iii) To use recruitment and outcomes data from the feasibility trial to estimate parameters for a future, definitive RCT and infer whether results show preliminary indications of potential efficacy.

This paper will report the intervention development process and the trial protocol, in accordance with recommended reporting guidelines.

## Methods

### Phase 1: intervention development and preliminary testing

#### Intervention development

The intervention was developed during phase 1 of the study, guided by the Medical Research Council (MRC) Framework for Developing Complex Interventions [20, 21] and building on the intervention delivered in the Zürich study [18, 19]. We developed the description of the intervention following the TIDIER (Template for Intervention Description and Replication) checklist [22].

A co-production group was convened. Some members of this group drew on their own lived experience of being detained under the Mental Health Act and/or supporting others so detained in contributing to the design and conduct of the study. Others were university-based researchers and clinicians with relevant experience, with some group members having more than one relevant role. The co-production group met at least twice monthly during the intervention development phase to support the co-design of the intervention, as further described below.

#### Inputs to intervention development

The following were the main inputs to intervention development, reviewed by the Co-Production Group through written summaries and presentations at the group’s meetings:

- Review of intervention content and experiences of engaging patients and staff in the Zürich study [18, 19]. As well as reviewing the published papers from Zürich, we engaged Dr Barbara Lay, lead author of the study, as a consultant member of our team (and co-author of this paper). She has thus been available to describe the team’s approaches and experiences in delivering the original intervention.
- Service user qualitative study: qualitative interviews were conducted with 20 people who had experienced compulsory detention, exploring their experiences and suggestions for averting detention. Interviews were carried out by members of the co-production group during the intervention development phase, and relevant data was rapidly extracted to inform the intervention development process. These interviews will be reported in full in a subsequent publication. Ethical approval was obtained for this preliminary study from the University College London (UCL) Research Ethics Committee (Ethics ID Number: 15249/002 (this was separate from NHS ethical approval subsequently obtained for the feasibility trial).
- Clinician qualitative study: as a second component in our preliminary study, we conducted qualitative interviews with 13 clinicians with experience in the process of detaining people under the Mental Health Act, or of working with people so detained. These interviews focused on clinician views about pathways leading to compulsory detention, and how this might have been averted.
- Review of published evidence and interventions: we reviewed evidence from peer-reviewed literature regarding effective and ineffective approaches to reducing detentions and obtained details of study interventions of interest to review their suitability for adaptation as part of our intervention. Sources included a systematic review of interventions aiming to reduce compulsory admissions through crisis plans and/or advance statements [13], a systematic review of evidence on the effectiveness of self-management for people with severe mental illnesses [23], and a systematic review of evidence on implementation on self-management interventions [24], as well as a previous self-management and crisis planning intervention developed through a co-production process by a team led by SJ and BLE [25].
- Guided discussions with co-production group: these included the following:
  o Discussion led by an experienced therapist and intervention developer (LW) to identify the values and qualities a Personal Mental Health Worker (PMHW) delivering the intervention should have.
  o Discussions led by members of the study team with relevant expertise on equalities and cultural appropriateness (PN, HMB) on how to make the intervention engaging, acceptable, and relevant across a range of cultural groups, especially those at high risk of compulsory admission.

**Fig. 1:**
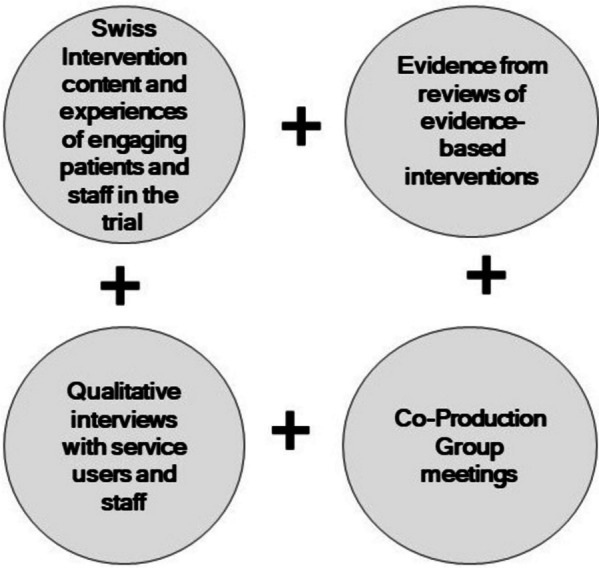
Intervention development diagram

Figure 1 outlines the key components for the development of the intervention.

#### Process of intervention development

Our co-production group consisting of nine service users and carers, five clinicians, and six researchers (some with multiple roles) met twice monthly through the development phase, reviewing all the above inputs, and making key decisions. The co-production group was diverse in terms of ethnic background, gender and age and all service users, carer, and clinician members had relevant personal or professional experience of the compulsory admission process.

A manual and content for a series of sessions was developed and iteratively reviewed by the co-production group, and by the study co-applicant team and the PMHWs recruited to deliver the intervention.

Key considerations identified by the co-production group as needing to be borne in mind throughout the development and delivery of the intervention included the following:

- The centrality of therapeutic engagement (building trust and promoting hope)
- The need to be flexible throughout the delivery of the intervention, individualising structure, and content of the intervention
- The need to consider and discuss the impacts of culture, ethnicity, and race, including the effects of experiences of racism within and outside the mental health care system
- The importance of working with different explanations of being sectioned and different ways of seeing mental health difficulties, adapting interventions to fit with service users’ ways of seeing their difficulties and their pathways to being detained.
- The need for awareness of the potential for triggering distress and difficult memories through the intervention sessions
- The value in engaging other supporters (e.g. family and friends, community care coordinators) in work such as the development and implementation of crisis plans, but with a requirement to establish and adhere to service users’ preferences regarding sharing of information with their networks.
- The value of awareness of wider community resources, including for specific ethnic, national, and religious communities, sexual minorities, or people who have had particular adverse experiences, and of signposting to such resources.
- The a need to consider accessibility throughout the development of interventions and materials.

### Intervention content and delivery

The intervention drafted through the above process consists of four initial sessions with a PMHW, followed by monthly check-in sessions for the remainder of a year. PMHWs are clinical psychologists or other qualified mental health professionals with experience in delivering structured interventions to people with significant mental health problems. PMHWs aim to complete as many of these initial sessions, each lasting up to an hour, as possible while participants are still inpatients, but continue the intervention in the community following discharge when needed.

The key activities which PMHWs aim to complete in the first four sessions with each intervention participant are the following:

- Development of a collaborative formulation to allow an understanding of the pathway by which the participant came to be compulsorily detained
- Creation of a personalised crisis plan, addressing early warning signs of a crisis and ways of responding, potentially including family, friends, and professionals from whom they feel they may be able to get support. This is in digital and/or paper form, according to preference. This includes an opportunity to develop a “Message to future self” in written or recorded form, which may include advice on how to manage an incipient crisis or remind them of important aspirations.
- Development of an advanced statement recording care and treatment preferences if they become unwell in the future.
- Exploration of aspirations and recovery goals for the future.

As engagement is a central aim, there is considerable flexibility in the order of delivery of these activities, and in whether some of them continue into the 11 monthly check-in sessions. These check-in sessions are initiated by the PMHW and are intended to involve providing support, continuing, or revisiting elements of the intervention, checking for identified early warning signs, and prompting responses to avert a crisis. These sessions are up to 45-min in duration. The main modes of delivery are face-to-face for the initial sessions and phone or video calls for subsequent check-ins, but with flexibility to accommodate individual preferences and circumstances: text and WhatsApp messaging are also used to maintain contact.

PMHWs receive an initial two sessions of training to learn how to deliver the intervention, as well as access to the draft manual. Training is delivered by a senior clinician, qualified members of the research team, and experts by experience from the co-production group. PMHWs also receive monthly supervision sessions by a senior clinician to address any queries or challenges they may experience during intervention delivery.

During the feasibility study, all PMHWs will record information on the content and delivery of the sessions, such as details of session length, location and types of crisis management strategies utilised (e.g. assessment, formulation, development of crisis plan) on a pre-defined database, including a bespoke fidelity checklist designed for recording of whether key elements of the intervention have been delivered. Participants are also invited to have therapy sessions recorded: this provides documentation for the patient and helps researchers to analyse how the intervention is being delivered and what modifications might be needed. However, given the engagement challenges in this study, recording is presented to participants as an optional addition to the study.

### Preliminary testing

A preliminary sample of *n* = 6 meeting the eligibility criteria was recruited towards the end of the intervention development phase to receive an initial draft version of the intervention. This allowed the intervention to be studied and refined in the light of initial experiences The participants in this phase and the PMHWs delivering the intervention were interviewed and minor refinements based on their experiences and suggestions were introduced to the structure and content of the intervention and to the manual and materials supporting it. Final refinements were made to the intervention in discussion with the co-production group, focusing especially on flexibility and personalisation.

### Phase 2: feasibility trial and embedded qualitative study

#### Feasibility trial

A feasibility trial of the intervention will be conducted, following established guidance on feasibility and pilot trials [26, 27]. This protocol has been written in accordance with the Standard Protocol Items: Recommendations for Interventional Trials (SPIRIT) checklist, please see the additional file. Health Research Authority (HRA) and NHS Research Ethics Committee (REC) approval has been granted by the London-Bromley Research Ethics Committee (IRAS: 300,671; Protocol number: 143180; REC reference: 21/LO/0734).

#### Setting

Participants will be recruited from acute psychiatric inpatient wards within the catchment areas of three NHS mental health Trusts in England; one in inner London, one in outer London, and one covering a mixture of non-metropolitan areas in the North-West of England. Each Trust is the provider of a range of acute and community psychiatric services in its local catchment area. Both London Trusts have highly ethnically diverse catchment areas.

### Participant eligibility criteria

Eligible participants will be current inpatients who:

- Have been compulsorily detained under Sect. 2 or Sect. 3 of the Mental Health Act during their current hospital admission (these sections allow for respectively 28 days detention for assessment or a renewable 6-month detention period for treatment);
- Are due to receive community mental health care locally post-discharge;
- Are aged 18 and above;
- Have the capacity at the time of recruitment to give informed consent to participation in the trial and to receive the study intervention.

Participants will be excluded if they (a) are already receiving an intensive psychosocial intervention that focuses on crisis reduction (b) have a diagnosis of dementia or a brain injury (c) do not speak sufficient English to take part without an interpreter.

There are no exclusions on the basis of mental health diagnosis. Our aim is to recruit 80 participants, at least half from ethnic groups who are at greater risk than White British patients of being compulsorily detained according to recent NHS data [3] (including people from all Black and Black Mixed backgrounds and Asian Pakistani and Asian Bangladeshi patients). We aim to recruit 30 participants from each London centre and 20 from the North-West England centre.

### Recruitment process

Participants will be recruited from acute mental health inpatient wards for adults. Methods used to recruit will include discussing the study with inpatient staff teams, individually and at ward meetings, and inviting them to introduce the study to potentially eligible participants, screening of caseloads by Trust staff with access to case notes, and distribution of advertising materials onwards and via local social media. Inpatient staff will be asked to consider the stage of service users’ recovery and likely capacity to understand the study purpose and requirements before approaching patients on our behalf. Posters will also be put up, and flyers distributed on recruiting wards to advertise the study, and the study discussed at community meetings for patients when there are opportunities. We will also promote the study on social media related to the trust and share advertising material with relevant local advocacy and voluntary services within the trust’s locality.

Participants who are willing to consider participating in the study and are deemed likely to be eligible by ward staff will be approached either by a research worker within the study team or by a Clinical Studies Officer (these are members of NHS Trust Research and Development teams who are embedded with inpatient teams for the purpose of identifying and recruiting study participants). Patients can also self-refer by contacting the research team directly via details on recruitment materials. Potential participants will be provided with a verbal overview of the study and given a copy of the participant information sheet. They will be given at least 24 h to consider whether they are interested in the study. If they remain interested in taking part after this, their capacity to give informed consent will be assessed (including the understanding of trial processes and the intervention), and participation will begin once written informed consent has been obtained by a researcher.

### Interventions

The *intervention group* will be offered the co-designed study intervention as described above, consisting of four initial sessions delivered approximately once a week, and subsequent monthly contacts up to a year post-randomisation. This support will be in addition to treatment as usual (TAU), during hospital admission and post-discharge.

The *control group* will be offered TAU, which is a standard practice for feasibility trials. This potentially includes multi-disciplinary care from staff including mental health nurses, nursing assistants, psychiatrists, pharmacists, occupational therapists, and psychologists in both the inpatient and community settings. TAU will also be offered to the intervention group.

### Allocation

Randomisation will take place following the completion of baseline measures. Researchers at UCL who are independent of the study will allocate participants via a computer-generated allocation sequence to either the intervention or control group in a 1:1 ratio using block randomisation stratified by site (3:3:2 ratio inner London: outer London: North-West England) and ethnicity (ethnic minority groups at higher risk of detention vs lower risk groups 1: 1 ratio).

Some of the study team will be unblinded so that they are able to deal with any issues with intervention delivery as they arise. Other members of the study team, such as the study research assistants who will be collecting follow-up outcomes, will be kept blind to treatment allocation as far as possible. The study team will liaise with the PMHWs, who will not be blinded, to minimise the likelihood that the blind researchers will be accidentally exposed to information about group allocation (known as ‘blind breaks’). Blinding will be monitored, and if any blind breaks occur, they will be systematically recorded.

### Sample size

We aim to recruit 80 participants in line with recommendations by Consolidated Standards of Reporting Trials (CONSORT) for a pilot randomised controlled trials (RCTs) and to randomised them to either intervention or control group [28]. This is deemed a sufficient sample to examine the primary aim of the study, which is assessing feasibility parameters to inform decisions about a future fully-powered confirmatory trial—we have considered the need to have sufficient data both about the sample as a whole and high-risk ethnic groups.

The statistical power of the current feasibility trial will result in 80% power (with a two-sided alpha of 5%) of detecting a reduction in rates of compulsory readmission from 50 to 20% following the recruitment of 80 participants. Such a large difference between arms is not expected, but the risk of detention in each group will inform the calculation of sample size and can also inform a calculation of how likely it is that a significant result would be achieved in a full study (see Analysis section below).

### Discontinuation/withdrawal of participants

When consenting to participate in the trial, participants are consenting to randomisation, assessments, intervention, follow-up, and data collection. Participants also consent to the research team continuing to collect data from medical records should they lose capacity at any point during the trial, unless this consent is withdrawn. A participant who is known to lack capacity at the time of a follow-up interview will not be contacted for further assessments, but data collected up to that point will be retained, and the team will continue to use data from the participant’s medical records unless a request to withdraw from the use of this data is received, in which case no further data will be collected. If a participant who has lost capacity regains it within the timeframe for interview assessments, they will be contacted to seek consent to carry these assessments out (this maximises the numbers who can be included despite the transient loss of capacity which is frequent among people with severe mental health problems).

### Outcomes

The primary goal will be to assess feasibility outcomes. We will also collect data on planned outcomes for a future randomised controlled trial of the intervention, allowing assessment of the feasibility of intervention delivery and trial processes, collection of data needed to inform a power calculation for a definitive trial, and a preliminary assessment of the likelihood of a positive result from a definitive trial.

### Feasibility outcomes

Detailed trial parameters will be recorded. These will include rates and routes of identification of potentially eligible participants at each site, recruitment and acceptance of randomisation, rates, and patterns of attrition from treatment and trial assessments, delivery of each intervention component, completion rates for individual outcome measures, rates of serious adverse events in each arm of the trial, and event rates for the planned primary trial outcome of compulsory readmission.

## Progression criteria

The trial parameters will be assessed against the following progression criteria:

- Recruitment within 9 months of 80 trial participants.
- At least 50% of these participants are from ethnic backgrounds associated with an elevated risk of being compulsorily admitted.
- At least 85% data completeness on primary outcome measure for trial (repeat compulsory admission within a year)
- At least 60% data completeness for secondary outcomes at 1 year
- At least 75% of intervention participants have developed a crisis plan and have received at least 3 intervention sessions.

If the above are achieved, we will assume that current protocols and procedures are suitable for a full trial, testing the hypothesis that the intervention reduces compulsory hospitalisation. If we fall short on any criteria by up to 20% we will consider whether improvements can be made in procedures and processes to make a full trial achievable. If we fall short by more than 20%, we will assume that a full trial is only justified if substantial changes are made and a further pilot takes place.

### Trial outcomes

Trial outcomes will be measured via clinical records and interviews with study researchers. Research interviews will be conducted at baseline prior to randomisation, and at 6 and 12 months after randomisation. A final follow-up point at 24 months will involve health record data only.

1. Primary trial outcome

The planned primary outcome for a future definitive trial is whether the participant has been compulsorily detained in a hospital under Sect. "Secondary outcomes" or Sect. 3 of the Mental Health Act within 1 year of randomisation. This data will be extracted from participants’ health records.

2. Secondary outcomes

From health records:

The following will be obtained from electronic data about patients held by Trusts:

- Compulsory admission within 24 months of randomisation.
- Whether participants remain engaged with services

### From research interviews

The remaining secondary outcome measures will be collected by a research assistant blind to treatment allocation. The following measures will be administered during a face-to-face, video, or phone interview (depending on service user preference, with face-to-face or video call preferred if possible):

- Satisfaction with services will be examined using the Client Satisfaction Questionnaire (CSQ) [29]. This is an 8-item scale where participants can rate their satisfaction with various aspects of their care on a 4-point Likert scale.
- Self-rated recovery will be measured by the 15-item Questionnaire about the Process of Recovery (QPR) [30]. Participants can score from 0 (disagree strongly) to 4 (agree strongly) on each item and score up to a maximum of 40 on the scale.
- Self-management confidence will be measured using the Mental Health confidence scale [31]. Participants report their confidence in managing their mental health for 16 items rated on a Likert scale from very non-confident to very confident.
- Quality of life will be measured by the REQOL-10 [32] and EQ-5D-5L [33]. They are both widely used measures in clinical trials with populations with severe mental health problems. On the REQOL, participants rate their quality of life on 10 items from 0 to 4. A participant can score a total of 40 and this information can be used to derive disability-adjusted life years (QALYs). For the EQ-5D-5L, we used only the physical health item, where participants can score from 1 (no problems) to 5 (severe problems). This information can be used to derive quality-adjusted life years (QALYs)
- Psychiatric symptoms will be assessed using the Brief Psychiatric Rating Scale (BPRS) [34]. Participants are rated by a researcher/research assistant on 18 items from 0/ NA (not assessed) to 7 (extremely severe) to give an overall score of psychiatric symptoms.

### Health economic analysis

Service use data will be collected using an adapted version of the Client Service Receipt Inventory (CSRI) [35]. Costs will be calculated by combining this information with appropriate unit costs. The costs of the intervention will be calculated from information on staff time and other requirements.

Descriptive data such as demographic and clinical/service user characteristics will also be recorded, including Community Treatment Order status, previous admission and compulsory detention history clinical diagnosis, and demographic data including age, sex, and ethnic group.

### Qualitative study

An embedded qualitative study, involving semi-structured interviews with participants in the experimental group and with the PMHWs delivering the intervention, will be used to investigate experiences and acceptability of the intervention and desirable modifications before proceeding to a full trial.

Semi-structured interviews will be conducted with up to 30 consenting intervention group participants: the size of the sample will be determined by when we appear to have attained thematic sufficiency and a sample that is representative in terms of demographics and diagnosis (or else may reflect the number of intervention group participants who are willing and able to give informed consent to an interview).

Interviews will be carried out between 6 and 9 months after recruitment, usually by service user researcher members of the co-production group to facilitate empathy and open disclosure, supported by the research team, or if not available, interviews will be carried out by a study research worker. Study researchers will interview the PMHWs who have delivered the intervention.

Topic guides will explore experiences of the intervention and its acceptability, barriers, and facilitators to making use of it, possible mechanisms of effect, potential benefits or harms, and suggested changes. Data collection and analysis will be guided by the Theoretical Framework of Acceptability [36] using thematic analysis, exploring in relation to the intervention the seven domains of acceptability specified in this framework: affective attitude, burden, perceived effectiveness, ethicality, intervention coherence, opportunity costs, and self-efficacy.

### Participant timeline

Intervention development took place between May 2021 and March 2022. The six preliminary patients were recruited in April and May 2022. For the feasibility study, baseline recruitment will be from May 2022 to the end of January 2023 with an estimated 10 participants recruited per month. Participants will be followed up 6 months and 12 months post-baseline to complete all measures again, and medical records will be assessed 24 months post-baseline.

Please see Table 1 (SPIRIT Table) which outlines the schedule of enrolment, interventions, and assessments.

**Table 1:** SPIRIT Table—schedule of enrolment, interventions and assessments

	Screening (pre-treatment assessment)	Intervention phase	6-month post-baseline (post-therapy)	12-month post-baseline (follow-up)	24-month post-baseline (follow-up)
	Day 1–7	Week 1–52	Week 24	Week 52	Week 104
Informed consent	X				
Eligibility confirmation	X				
Randomisation	X				
Compulsory admission				X	X
CSQ	X		X	X	
QPR	X		X	X	
REQOL	X		X	X	
MHCS	X		X	X	
CSRI	X		X	X	
Intervention delivery		X			
Qualitative interview (intervention arm participants only)			X^a^		

^a^Will be conducted 6–9 months post-randomisation

## Data management, security and analysis

### Data management and security

All staff have been trained in and will adhere to the requirements of the General Data Protection Regulation (GDPR) (2016/679) and the UK Data Protection Act (2018) with regard to the collection, storage, processing, and disclosure of personal information, and will uphold the Act’s core principles.

Identifiable information (name, address, contact details, and GP name) will be stored securely and separately from other study data on a password-protected database. The Case Report Forms (CRFs) will not bear the participant’s name or other personally identifiable data. Data on service use, psychiatric diagnosis, and related symptoms, risk assessment, and hospitalisation will be extracted from Trusts’ electronic records. All information extracted from medical notes will be directly added to the CRF and anonymised through the use of a participant number.

All electronic data collected during the study will be stored on a password-protected REDCAP database, which is a secure web application for building and managing online databases. The database is password protected and only the trial manager and researchers recruiting and enrolling participants in the study have access.

Participants are made aware at the time of recruitment that if a participant discloses a serious risk of harm to themselves or others, researchers would consider breaching participants’ confidentiality to share the necessary information with the relevant clinical team or, if the urgency of the situation did not allow prior discussion with clinical services, the emergency services. However, this would be judged on a case-by-case basis, and confidentiality would only be breached for serious disclosures where there is considerable concern about immediate risk to self or others.

## Data analysis

### Statistical analysis

A separate Statistical Analysis Plan will be pre-specified which will be held within the Statistics Master File. It will contain following sections: (I) Introduction, (ii) study methods, (iii) statistical principles, (iv) trial population, (v) analysis of primary and secondary outcomes). We will register this SAP in a public-facing database (e.g. clinicaltrials.gov) prior to unblinding of the statistician and the commencement of data analysis. The current trial statistician (RG) and oversight statistician (NF) remain blinded to intervention allocation during the conduct of the trial and shall remain so until after the end of data collection.

### Feasibility outcomes

The primary aim of the study is to assess the feasibility of recruiting, randomising, and retaining 80 participants in the trial. Descriptive statistics will include the rates of identification of potentially eligible participants, recruitment and acceptance of randomisation, rates, and patterns of attrition from treatment and trial assessments, delivery of each intervention component, completion rates for individual outcome measures, and event rates on the outcome of compulsory readmission.

### Trial outcomes

The analysis population will consist of all eligible patients who have given consent and have been randomised to an intervention arm irrespective of the completion of the study. We will use an intention-to-treat analysis.

For binary and continuous trial outcome variables, we will assess characteristics of the statistical distribution, enabling us to power a larger subsequent RCT.

### Sub-group analyses

Subgroup analyses are not envisaged especially given the relatively restricted sample size and the resultant lack of power for multiple analyses.

### Inferential analyses

This pilot study will not have sufficient statistical power to assess the effectiveness of the intervention but will allow an assessment of whether the direction and magnitude of any effect found for the proposed primary outcome are consistent with a hypothesis that the programme is effective in reducing repeat detentions. For this reason and to test the analysis envisaged for a future, fully powered, effective RCT, primary outcome at follow will be compared between study arms, using appropriate multi-level models. Our planned analysis is hierarchical multi-level modelling, allowing for the clustering of residuals between centres by introducing the variable coding for centre as a random intercept. We will enter all other stratification variables as fixed effects. Other potential baseline covariates (e.g. age, ethnicity) will be entered in serial adjusted models where relative fit will be assessed using Akaike and Bayesian Information Criteria. The goodness of fit will be determined using Hosmer Lemeshow test of fit. We will assess the robustness of the results to missing data by carrying out sensitivity analyses in which the missing data are assigned extreme values to determine the effect of potential extreme missing values on the stability of the reported results. We will assess the effect of time by analysing results with time as a random slope variable.

### Missing data

Patterns of missingness in recorded data will be reported as part of the feasibility outcome of the ability to retain participants in the study. Reasons for missingness will be investigated and reported We will conduct threshold analyses to describe the extent to which any missing data could make a qualitative difference to the result by imputing a poor outcome for all intervention group missing subjects, and an average one for all control group missing subjects, and vice versa. We will report the outcome of these statistical investigations where appropriate.

### Health economic analysis

Service use data will be described in each arm to inform future analysis. QALYs will be derived from measures of quality of life and correlations between these, and other outcomes will be calculated. This will inform the choice of QALY measures for a future trial.

## Monitoring for adverse events

Both groups will be monitored for the occurrence of adverse events (AEs) and a log kept. The severity of adverse events is assessed by several categories, where severe adverse events are those that result in alteration, discomfort, or disability which is clearly damaging to health. All serious adverse events (SAEs) will recorded in a trial log and assessed by the chief investigator (CI) and site principal investigators as likely to be related to the intervention (a causal relationship between the intervention and an adverse event is at least a reasonable possibility, i.e. the relationship cannot be ruled out); not related (there is no reasonable possibility of a causal relationship between the intervention and an adverse event); not assessable (unable to assess on information available).

All serious AEs will be recorded in the medical records, the CRF, and the sponsor’s AE log. The log of SAEs will be reported to the sponsor twice per year. Where the event is unexpected and thought to be related to the intervention, this will be reported by the Investigator to the Health Research Authority within 15 days. Completed SAE forms must be sent within 5 working days of becoming aware of the event to the Sponsor (the UCLH/UCL Joint Research Office, acting on behalf of UCL).

## Oversight

The CI (SJ) and co-CI (BLE) will be responsible for the day-to-day monitoring and management of the study. They will ensure there are adequate quality and number of monitoring activities conducted by the study team. This will include adherence to the protocol, procedures for consenting and ensuring adequate data quality.

An independent trial steering committee (TSC), including a representative of the funder, clinicians, researchers, a lived experience participant, and a statistician, will meet regularly to provide independent oversight of trial progress, safety, and analysis plans.

A Data Monitoring Committee (DMC) is not planned for this pilot trial, which does not collect interim data and has no pre-planned stopping criteria. The study management team will ask the steering committee to review this plan before the start of the trial in the second year of the study and will recruit a DMC if the TSC advises this.

## Discussion

The proposed study is innovative in that the evidence based on the important question of how to prevent compulsory detention has remained surprisingly limited, despite consensus among service users, carers, clinicians, and policymakers that this should be the priority. Our main goal in the current study is to assess the feasibility and acceptability of intervention delivery and implementation of trial processes. This is especially necessary in view of challenges in delivering research with this population, where there are challenges associated both with conducting research with people whose mental health problems may be acute and severe and with the many reasons people may have to mistrust services and professionals following their experiences of compulsory detention. We hope that the active engagement in our study of research team members with relevant personal experience will help us to overcome such barriers. The paucity of previous research and the difficulty implementing interventions as hoped in those studies that have been conducted may reflect such barriers: the selection of an intervention in which considerable attention has been to achieving continuing participation in implementing crisis planning through monthly contacts, and the co-design process are intended to address these barriers.

The main reported outputs from the current studies will be an assessment of feasibility and acceptability by quantitative and qualitative means, but decisions about progressing to a definitive study will also be informed by the feasibility and acceptability of collecting the proposed trial outcome studies, by the data obtained to inform a power calculation, and by assessment from our results of the likelihood of obtaining a positive result in a definitive trial. Feasibility and acceptability among people from minority backgrounds associated with a greater risk of compulsory detention will also be a key consideration in the design of a further trial.

### Supplementary Information

**Additional file 1.** The TIDieR (Template for Intervention Description and Replication) Checklist*.

**Additional file 2.**

**Additional file 3.** SPIRIT 2013 Checklist: Recommended items to address in a clinical trial protocol and related documents*.

**Additional file 4. **Examining the feasibility and acceptability of a new crisis-planning intervention for those who have been “sectioned” under the Mental Health Act.

## Data Availability

This is a protocol paper and thus does not report any data. When the study is completed, the data will be available from the corresponding author on reasonable request following completion of the main analyses.

## Abbreviations

AEs: Adverse events
BPRS: Brief Psychiatric Rating Scale
CRF: Case report form
CI: Chief Investigator
CSQ: Client Satisfaction Questionnaire
CSRI: Client Service Receipt Inventory
CONSORT: Consolidated Standards of Reporting Trials
DMC: Data Monitoring Committee
GDPR: General Data Protection Regulation
HRA: Health Research Authority
MRC: Medical Research Council
NIHR: National Institute of Health Research
REC: NHS Research Ethics Committee
PMHW: Personal Mental Health Worker
QPR: Process of Recovery Questionnaire
QALYs: Quality-adjusted life years
RCTs: Randomised controlled trials
SAE: Serious Adverse Event
TIDIER: Template for Intervention Description and Replication
TAU: Treatment as usual
TSC: Trial Steering Committee
UCL: University College London
