# Supplementary material for: A crisis planning and monitoring intervention to reduce compulsory hospital readmissions (FINCH study): protocol for a randomised controlled feasibility study
Source: Pilot Feasibility Stud. 2024 Feb 20;10:35. doi: 10.1186/s40814-024-01453-z (PMC10877855; doi:10.1186/s40814-024-01453-z)
Supplement: Supplementary file 2 — Additional file 2. [file 40814_2024_1453_MOESM2_ESM.docx]

[
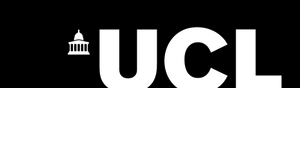
](https://www.google.com/url?sa=i&rct=j&q=&esrc=s&source=images&cd=&ved=2ahUKEwiGxuLNrZHkAhXNSBUIHTfyA7kQjRx6BAgBEAQ&url=https://www.ucl.ac.uk/&psig=AOvVaw1R6UVOQD4TxF5_wG9hc50S&ust=1566387313858386)

Participant Identification Number for this study: ………. IRAS ID: 300671

**Please complete this form after you have read the Information Sheet and/or listened to an explanation about the research.**

**Title of Project: Examining the feasibility and acceptability of a new crisis-planning intervention for those who have been sectioned under the Mental Health Act** *(Service User Participant, Pilot Trial)*

**Name of Researcher:** _________________

Thank you for considering taking part in this research. The person organising the research must explain the project to you before you agree to take part. If you have any questions arising from the Information Sheet or explanation already given to you, please ask the researcher before you decide whether to join in. You will be given a copy of this Consent Form to keep and refer to at any time.

I confirm that I understand that by ticking/initialling each box below I am consenting to this element of the study. I understand that it will be assumed that unticked/initialled boxes means that I DO NOT consent to that part of the study. I understand that by not giving consent for any one element, except where indicated, that I may be deemed ineligible for the study.

|  |  | Tick box |
| --- | --- | --- |
| 1. | I confirm that I have read the information sheet dated **04/10/22** (version 4) for the above study. I have had the opportunity to consider the information, ask questions about it and have had these questions answered satisfactorily. |  |
| 2. | I understand that my participation is voluntary and that I am free to withdraw at any time without giving any reason, without my medical care or legal rights being affected. |  |
| 3. | I agree that a researcher may contact me at subsequent points during the next year to see whether I am willing to take part in other data collection activities related to this study. I am aware that when they contact me, I can decide whether to take part or not. |  |
| 4. | I consent to the research team having access to my medical notes to record research involvement (therapy and assessment sessions), key risk issues and adverse events, and check my contact with NHS services over the next 24 months. |  |
| 5. | I agree to my GP (General Practitioner) and the mental health service which supports me being informed of my participation in the study. |  |
| 6. | I understand that data collected during the study may be looked at by individuals from University College London, from regulatory authorities or from the NHS Trust, where it is relevant to my taking part in this research. I give permission for these individuals to have access to my data. |  |
| 7. | I understand that, if I withdraw from the study, unless I tell the research team to delete data which has already been collected, they will continue to use this data and collect information about me from my patient records, but will not contact me any more about the study. |  |
| 8. | I consent to a copy of this consent form being kept with my medical notes. |  |
| 9. | I understand the potential risks of participating and the support that will be available to me should I become distressed during the course of the research. |  |
| 10. | I understand the direct/indirect benefits of participating. |  |
| 11. | I understand that the data will not be made available to any commercial organisations but is solely the responsibility of the researcher(s) undertaking this study. |  |
| 12. | I understand that I will be offered a £20 gift in vouchers or £20 in cash for my participation in each research assessment, once I have taken part in it, but that I will not benefit financially in any other way from this study or from any possible outcome it may result in in the future. |  |
| 13. | I understand that the information I have submitted will be published as a report. All data will be anonymised and the report will not include any personally identifying details. |  |
| 14. | I am aware of who I should contact if I wish to lodge a complaint. |  |
| 15. | I understand that the data I provide will be archived at UCL at the end of the study. |  |
| 16. | **I agree that my anonymised research data may be used by others for future research at UCL. [No one will be able to identify you when this data is shared.] (optional)** |  |
| 17. | **I wish to receive a copy of a report detailing the findings of the study (optional).** |  |
| 18. | **I consent to my crisis-planning therapy sessions being audio-recorded (optional).** |  |
| 19. | **I consent to being contacted about the results of the study (optional).** |  |
| 20. | **I consent to being contacted by a researcher about taking part in a qualitative interview about my experiences of the intervention and research (optional).** |  |
| 21. | I voluntarily agree to take part in this study. |  |

______________________ ________________ ____________________

Name of Participant Date Signature

______________________ ________________ ____________________

Researcher Date Signature

1 copy for participant; 1 for researcher; 1 to be kept with medical notes
