# Supplementary material for: A crisis planning and monitoring intervention to reduce compulsory hospital readmissions (FINCH study): protocol for a randomised controlled feasibility study
Source: Pilot Feasibility Stud. 2024 Feb 20;10:35. doi: 10.1186/s40814-024-01453-z (PMC10877855; doi:10.1186/s40814-024-01453-z)
Supplement: Supplementary file 4 — Additional file 4. Examining the feasibility and acceptability of a new crisis-planning intervention for those who have been “sectioned” under the Mental Health Act. [file 40814_2024_1453_MOESM4_ESM.docx]

[
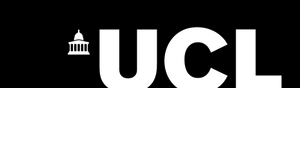
](https://www.google.com/url?sa=i&rct=j&q=&esrc=s&source=images&cd=&ved=2ahUKEwiGxuLNrZHkAhXNSBUIHTfyA7kQjRx6BAgBEAQ&url=https://www.ucl.ac.uk/&psig=AOvVaw1R6UVOQD4TxF5_wG9hc50S&ust=1566387313858386)

**Examining the feasibility and acceptability of a new crisis-planning intervention for those who have been “sectioned” under the Mental Health Act**

**INFORMATION FOR PARTICIPANTS**

**Finch Study Pilot Trial Participant Information Sheet****_V4_04/10/22**

This information sheet is to let you know about a research study that you may be able to take part in if you wish. Before you decide, it is important that you understand why the research is being done and what it would involve for you. The researcher will go through the information sheet with you and answer any questions you have. You can also talk to others about the study if you wish, and please ask us if there is anything that is not clear.

This study has been reviewed by an NHS ethics committee to ensure that the rights, safety, dignity and well-being of everyone that takes part in this study are protected. REC reference: 21/LO/0734.

**Principal Investigators (lead researchers)**

Professor Sonia Johnson ([s.johnson@ucl.ac.uk](mailto:s.johnson@ucl.ac.uk))

& Dr Bryn Lloyd-Evans ([b.lloyd-evans@ucl.ac.uk](mailto:b.lloyd-evans@ucl.ac.uk))

Division of Psychiatry, University College London, 6^th^ Floor, 149 Tottenham Court Road, London, W1T 7NF

**What is the purpose of this study?**

The number of people being involuntarily admitted to mental health hospitals under the Mental Health Act (being “sectioned”) has been increasing in the UK. One way to try and reduce this number is to offer support to people who get “sectioned” to try and reduce the likelihood of it happening again in the future.

There has not been very much research carried out to understand what types of support might help stop or reduce the likelihood of people being “sectioned”. The aim of this study is to develop and test a new type of support that aims to reduce the likelihood of sectioning happening again once someone leaves hospital. This support will involve developing a crisis plan and regular contact from a psychologist over the next year to help people develop skills to manage their own mental health and respond if another crisis may be developing. In this study, we are particularly keen to include and consider how to help people from ethnic minority backgrounds as they are more likely to be “sectioned” compared to White British people.

This part of research study is looking for people who are willing to participate in a pilot trial, where half the people taking part are allocated by chance to be offered our new type of support, in addition to their usual care; while the other half are not offered the new support but continue to receive their usual care. We will find out whether it is possible to deliver the new type of support and test it in a research trial. We will hear from people taking part whether the new support is acceptable and feels helpful, and we will get a first indication about whether it may be effective in helping to reduce the likelihood of people being “sectioned” again in the future.

Why have I been given this information?

You have been given this information because you have been recently “sectioned” under the Mental Health Act (section 2 or 3) and are due to receive community mental health care locally following discharge. You are aged over 18 and have capacity to consent at the time of recruitment.

**Do I have to take part?**

No. Taking part in this research study is voluntary therefore it is up to you to decide whether or not to take part. There is no obligation for you to take part and if you decide not to take part, this will have no effect on your current care and support from health and social care services. If you do decide to take part, you will be asked to sign a consent form. Even after signing the consent form you will still be free to withdraw at any time and without giving a reason. You can choose to simply let the researchers know that you no longer wish to take part. This will not affect any care you may receive in the future.

**What will happen if I take part?**

You can meet a researcher for this study at a convenient location for you to discuss the study in more detail. The researcher will talk through the study in detail with you, explaining the reasons for running this study and answering any questions you may have. If you are interested in taking part you will be asked to sign a consent form.

The following section will explain what will happen in the study if you choose to take part.

***Meetings with researcher***

All people taking part in the study (whether or not you are allocated by chance to be offered our new type of support) will be asked to meet with a researcher three times over the next 12 months. We will ask you for the following details that are best to contact you on, so that the researcher can contact you to meet with over the 12 months: telephone numbers, email, home address, telephone number of family member of friend (You can decide to give us more than one contact detail). Your medical records will also be assessed at 24 months, but you will not need to meet with the researcher at this point, the researcher will need to obtain your NHS Number from the ward team to be able to access your medical notes.

The three meetings will take place following this timeline:

- **Meeting 1:** Start of the study
- **Meeting 2:** Six months from the start
- **Meeting 3:** Twelve months from the start

Meeting 1 will take place on the hospital ward, Meetings 2 and 3 will take place either remotely (via video or telephone), or at your local community mental health service or at your home address (you will get to decide what suits you best).

These meetings will last for approximately 60 minutes each. You will be asked to complete some questionnaires about your mental health recovery and your views on the NHS services you are receiving. If you take part in the crisis-planning support, you may also be invited to an in-depth interview where you will be asked questions about your experiences of receiving this support. This in—depth interview would be additional to completing the questionnaires, and would take up to an additional 60 minutes. If we wish to invite you to this additional in-depth interview, we will contact you separately and you can choose then whether you want to take part in this.

***Randomisation***

After the first meeting with a researcher, the study researcher will let you know whether you have been allocated by chance (“randomised”) to receive the new crisis-planning support intervention in addition to your usual care, or to continue to have your normal treatment without receiving the new type of support. This means that not everyone who is taking part in the study is receiving the new type of support. If you choose to participate in the study, you will have a 50:50 chance of either receiving the new support in addition to your usual care, or continuing with treatment as usual. A computer will allocate you by chance to one group or the other. This is the best way to compare people who are receiving the new support to those who are not, so that we can make conclusions about it.

***The new type of support***

If you are randomly allocated to the group of people who will be offered the new type of support in addition to your usual care, it will consist of the following:

- Four individual sessions with a personal mental health worker (who will be a clinical psychologist or another member of staff with equivalent skills). These sessions will focus on discussing your risk factors for relapse, providing information about treatment and services and exploring your recovery goals. You will receive these sessions on the hospital ward usually but they may take place outside the ward, if you are discharged, or you may also communicate with the personal mental health worker over the phone or using video-calling software.
- You will work with the personal mental health worker to create an individualised crisis plan, which you can use once you have been discharged from hospital.
- We will ask for your consent to audio-record these four sessions. This is so that we can check that the personal mental health worker is delivering the new type of support in the same way for everyone. Your consent is optional, so you do not have to agree to these sessions being recorded to take part in the study.
- You will be offered a call from the personal mental health worker each month over the next 11 months (either on the telephone or using video-calling software). You will discuss how you are coping and how you have been applying the plan in your life and whether it needs any changes.

***Treatment as Usual***

If you are not randomly allocated to receive the new crisis-planning type of support, you will continue with your usual treatment. This will include your current care and treatment in hospital, and whatever support and treatment the NHS has arranged for you when you leave hospital.

***Study Diagram***

The following diagram shows the different stages and timings in this study:

**Meeting One *(questionnaire measures)***

**Meeting 1**

You will be randomly allocated to either receive the crisis-planning intervention (in addition to usual treatment) or continue with treatment-as-usual

Crisis-planning intervention (4 sessions) + 11 monthly follow-up calls with personal mental health worker

Also continue to receive treatment as usual

**1**

Treatment as usual

**Meeting Two *(questionnaire measures)***: 6 months later

(Some people from the intervention groups will also be invited to an interview at this point)

**Meeting 1**

**Meeting 1**

**Meeting Three *(questionnaire measures)***: 12 months later

**Meeting 1**

At 24 months, the research team will collect data on your service use. You will not be required to meet with the team again at this point.

**Will taking part in the study cost me anything?**

No. The study will only involve your time.

**Who will know I am participating in the study?**

Other people involved in your care such as your Consultant Psychiatrist and Care Coordinator will be informed that you are participating in this study as we will record your sessions with the research and personal mental health worker on your clinical notes. This is to ensure your wider care team are updated about you research involvement. We will also send a letter to your GP to inform them of your participation in the study.

If at any time you disclose something to us that relates to yourself or someone else being in significant risk of harm, we would share this with your clinical team and relevant services if needed (e.g. emergency services).

**How will we use information about you?**

We will need to use information from you for this research project. This will include your name and contact details, and any data you give us through taking part in this research study (e.g. questionnaire data and data from your medical records). Everyone involved in this study will keep your data safe and secure. People who do not need to know who you are will not be able to see your name or contact details. We will use study code numbers, rather than your name, to refer to you. This is to keep your name and any identifying details anonymous and confidential. We will also follow privacy rules. Once we have finished the study, we will keep some of the data so we can check the results. We will write our reports in a way that no-one can work out that you took part in the study.

The research team will need access to your clinical notes in order to collect information on your contact with services (e.g. home treatment teams and inpatient care), and on any updates about your safety (e.g. difficulties with suicide and self-harm) during the 24-month data collection period, which starts when you enter the trial. The researchers will also need to ask your mental health team to update your clinical notes when you have a research meeting or take part in a crisis-planning therapy session. This is to ensure your wider care team are updated about your research involvement.

The results of this research study and any published versions will also be anonymous; your name will not be quoted and you will not be individually identified in any reports or publications from the study. You can ask to receive a copy of the results of the study but we cannot provide individual results. We will send you a brief report of the findings of the study.

All completed questionnaires will be stored securely at participating NHS Trusts or at UCL in locked filing cabinets in secure offices. At the end of the study, all paper documents will be transferred to UCL for archiving. Completed questionnaires will also be kept in a separate locked filing cabinet to any documents with personal information on them (such as your name). If you are completing questionnaires outside of hospital (e.g. during a home visit) these will immediately be taken to UCL or the relevant NHS Trust and the storage procedures outlined above will be followed.

All electronic and audio data (such as answers to questionnaires typed into a computer file or recordings of your sessions with the mental health worker, if you consent) will be stored on a password protected computer which only the researcher will have access to. All data and personal information from this study will be kept for ten years after the study has finished so the study can be written up for publication in a research journal as recommended by the Data Protection Act (2018). After this point, all data will be destroyed.

**What are your choices about how your information is used?**

- You can stop being part of the study at any time, without giving a reason, but we will keep information about you that we already have.
- If you choose to stop taking part in the study, we would like to continue collecting information about your health from central NHS records for the entire study duration (24 months). If you do not want this to happen, tell us and we will stop. We need to manage your records in specific ways for the research to be reliable. This means that we won’t be able to let you see or change the data we hold about you.
- You can decide whether or not you would like your anonymised research data to be used by others at UCL for future research. This is completely optional.

**Where can you find out more about how your information is used?**

You can find out more about how we use your information:

• at [www.hra.nhs.uk/information-about-patients/](http://www.hra.nhs.uk/information-about-patients/)

• our leaflet available from [the ward or via email – [m.birken@ucl.ac.uk](mailto:m.birken@ucl.ac.uk)]

• by asking one of the research team

• by sending an email to Dr Mary Birken, the senior study researcher – [m.birken@ucl.ac.uk](mailto:m.birken@ucl.ac.uk)

• by sending an email to data-protection@ucl.ac.uk

The data custodian for this study is Professor Sonia Johnson.

**What are the advantages and disadvantages of taking part?**

If you are allocated to receive the new crisis planning support from a personal mental health worker, we hope you will find this helpful. If you are allocated to the group of people who are not offered our new type of support, you may be disappointed, but you will continue to receive your usual care just the same. Whichever group you are part of, you will be contributing to developing and testing a new way of working which may go on to help other patients in the future.

If you are allocated to receive the new crisis planning types of support, then during the sessions with the personal mental health worker, you will discuss a range of topics which might be sensitive. Completing the questionnaires with researchers will take up some of your time and may feel burdensome. This will include discussing events prior to you getting “sectioned” and thinking about things which may be difficult in future when you leave hospital. It is possible that talking about your personal experiences could sometimes lead to feeling upset. The personal mental health worker will be sensitive to your needs as they have experience working with people with upsetting or distressing emotions. You are free to withdraw from the research study at any point and this will not affect the on-going care you are currently receiving.

You will be given £20 in vouchers or £20 cash as a token of appreciation for each assessment interview you complete.

**What happens if something goes wrong?**

This project does not have any medical interventions such as asking you to take a new medication. You will only be asked to complete questionnaires when meeting with the researcher. If you are randomly allocated to receive the crisis-planning type of support, you will receive these sessions and you might also be interviewed about your experiences of the crisis-planning support.

We will do our best to keep all information that you give us confidential, However, if we obtain information that makes the research team concerned that there is a serious risk to you or someone else we will need to consider passing this on even if you have not agreed to this. Usually we would communicate this to NHS mental health services or your GP. In a rare situation where we felt there was an imminent risk of serious harm to someone else, we may need to contact police or emergency services.

It is necessary for us to point out that if you were to feel that taking part in this research project caused you upset or harm, there are no arrangements in place for offering compensation.

However, if you have any concerns about the way you have been treated during the course of the research, the researcher will be very happy to discuss this with you. You could also contact the Chief Investigators, whose contact details are above. If you wish to complain formally, or have any unresolved concerns about any aspect of the way you have been approached or treated during the course of this study, you can contact your local NHS Advice and Complaints Service:

Advice and Complaints Service

[**To be updated based on location** Camden and Islington NHS Foundation Trust

FREEPOST 1st Class (LON 12613)

London

NW1 0YT

Tel: 020 3317 3117

E-mail: [complaints@candi.nhs.uk](mailto:complaints@candi.nhs.uk)]

If at any point during the study you disclose any information that relates to yourself or someone else being at risk, we would inform your clinical team.

**What happens if I lose the ability to make informed choices (capacity) about the research during the study?**

If you lose capacity during the study, the research team will not collect any further information from you via any further research meetings while you are not able to make an informed choice about continuing to take part in the study. We will however keep information we have already collected from you with your consent, unless you ask us not to. We will continue to collect data about you from your health records, if you have already given us your consent to do this – unless you ask us not to. We will monitor your capacity to consent by liaising with your clinical team and will try to give you the opportunity to fully re-enter the study if you regain capacity.

**Minor Complaints**

If you take part in this project and later have a minor complaint then please contact Mary Birken in the first instance: [m.birken@ucl.ac.uk](mailto:m.birken@ucl.ac.uk)

**Formal Complaints**

If you continue to have concerns, please contact Professor Sonia Johnson, the Principal Researcher of this project: [s.johnson@ucl.ac.uk](mailto:s.johnson@ucl.ac.uk) . If you are still not satisfied, complaints may ultimately be referred the NHS Trust’s complaints team at [Insert local trust’s complaints team details].

**Independent Advice**

If you would like independent advice about taking part in research please contact your local Patient Advice and Liaison Service (PALS). You can get in touch with your local PALS by [insert local PALS details here].

**Local Data Protection Privacy Notice**

The controller for this project will be University College London (UCL). The UCL Data Protection Officer provides oversight of UCL activities involving the processing of personal data, and can be contacted at data-protection@ucl.ac.uk. This ‘local’ privacy notice sets out the information that applies to this particular study. Further information on how UCL uses participant information can be found in our ‘general’ privacy notice. For participants in research studies, click here: https://www.ucl.ac.uk/legal-services/privacy/ucl-general-research-participant-privacy-notice

The information that is required to be provided to participants under data protection legislation (GDPR and DPA 2018) is provided across both the ‘local’ and ‘general’ privacy notices.

The categories of personal data used will be as follows:

- Name (on consent form only)
- NHS Number
- You can decide to give us more than one contact detail listed below:
- Mobile phone number
- Landline number
- Home address
- Email address
- Telephone number of family member or friend
- Address to send report if wished,
- Gender
- Age
- Ethnicity
- Education
- Housing Status
- Living Situation
- Marital Status
- Sexual Orientation
- Employment Status
- Mental Health Diagnosis

The lawful basis that will be used to process your personal data are: ‘Public task’ for personal data and ‘Research purposes’ for special category data. Your personal data will be processed so long as it is required for the research project. We will endeavour to minimise the processing of personal data wherever possible. If you are concerned about how your personal data is being processed, or if you would like to contact us about your rights, please contact UCL in the first instance at [data-protection@ucl.ac.uk](mailto:data-protection@ucl.ac.uk).

**What is the next step?**

If you would like to take part, please contact the study team via our email address: [dop.finch@ucl.ac.uk](mailto:dop.finch@ucl.ac.uk) or please speak to your key worker.

**Thank you for reading this information sheet.**
